# Impact of alternative diagnostic labels for melanoma in situ on management choices and psychological outcomes: protocol for an online randomised study

**DOI:** 10.1136/bmjopen-2024-089558

**Published:** 2024-12-20

**Authors:** Zhuohan Wu, Brooke Nickel, Farzaneh Boroumand, David Elder, Peter M Ferguson, Richard A Scolyer, Blake O'Brien, Raymond Barnhill, Adewole S Adamson, Alexander C J van Akkooi, Jon Emery, Lisa Parker, Donald Low, Cynthia Low, Elspeth Davies, Sherrie Liu, Stacey Lewis, Bella Spongberg-Ross, Katy JL Bell

**Affiliations:** 1 Wiser Healthcare collabration, Sydney School of Public Health, Faculty of Medicine and Health, The University of Sydney, Sydney, New South Wales, Australia; 2 Sydney School of Public Health, Faculty of Medicine and Health, University of Sydney, Sydney, New South Wales, Australia; 3 Wiser Healthcare Research Collaboration, Sydney, New South Wales, Australia; 4 The University of Sydney, Sydney, New South Wales, Australia; 5 Department of Pathology and Laboratory Medicine, Hospital of the University of Pennsylvania, Philadelphia, Pennsylvania, USA; 6 Melanoma Institute Australia, North Sydney, New South Wales, Australia; 7 Tissue Pathology and Diagnostic Oncology, Royal Prince Alfred Hospital and NSW Health Pathology, Sydney, New South Wales, Australia; 8 Surgical Pathology, Sullivan Nicolaides Pathology, Brisbane, Queensland, Australia; 9 Department of Translational Research, Paris Sciences and Lettres Research University, Paris, France; 10 Department of Internal Medicine, Austin, Austin, Texas, USA; 11 The University of Texas at Austin Dell Medical School, Austin, Texas, USA; 12 General Practice and Primary Care Academic Centre, University of Melbourne, Carlton, Victoria, Australia; 13 Evidence, Policy and Influence Collaborative (EPIC), Charles Perkins Centre, University of Sydney, Sydney, New South Wales, Australia; 14 Cancer Voices New South Wales, Sydney, New South Wales, Australia; 15 Patient Researcher, Cambridge, Cambridge, UK; 16 Health Consumers New South Wales, Sydney, New South Wales, Australia

**Keywords:** Dermatological tumours, Adverse events, Clinical Decision-Making, Surgical dermatology, Surgical pathology, Patient-Centered Care

## Abstract

**Introduction:**

A diagnosis of melanoma in situ presents negligible risk to a person’s lifespan or physical well-being, but existing terminology makes it difficult for patients to distinguish these from higher risk invasive melanomas. This study aims to explore whether using an alternative label for melanoma in situ may influence patients’ management choices and anxiety levels.

**Methods and analysis:**

This study is a between-subjects randomised online experiment, using hypothetical scenarios. Following consent, eligible participants will be randomised 1:1:1 to three labels: ‘melanoma in situ’ (control), ‘low-risk melanocytic neoplasm’ (intervention 1) and ‘low-risk melanocytic neoplasm, in situ’ (intervention 2). The required sample size is 1668 people. The co-primary outcomes are (1) choice between no further surgery or further surgery to ensure clear histological margins greater than 5 mm and (2) choice between patient-initiated clinical follow-up when needed (patient-led surveillance) and regular routinely scheduled clinical follow-up (clinician-led surveillance). Secondary outcomes include diagnosis anxiety, perceived risk of invasive melanoma and of dying from melanoma and management choice anxiety (after surgery choice and follow-up choice). We will make pairwise comparisons across the three diagnostic label groups using regression models (univariable and multivariable).

**Ethics and dissemination:**

The study has been registered with the Australian New Zealand Clinical Trials Registry (ACTRN12624000740594). Ethics approval has been received from The University of Sydney Human Research Ethics Committee (2024/HE000019). The results of the study will be published in a peer-reviewed medical journal, and a plain language summary of the findings will be shared on the Wiser Healthcare publication page (https://www.wiserhealthcare.org.au/category/publications/).

**Trial registration number:**

Australian New Zealand Clinical Trials Registry (ID 386943).

**STRENGTHS AND LIMITATIONS OF THIS STUDY**

- The randomised design enables robust comparison of diagnostic labels on decision-making and psychological outcomes.
- The study has been co-designed with patients, members of the public and clinicians to ensure that labels and evidence are relevant to end-users.
- The large online randomised study is representative of adults in the Australian community.
- The study’s hypothetical nature limits its ability to capture real patients after an actual melanoma in situ diagnosis (or alternative label).
- The study does not explore the potential for recalibration of diagnostic thresholds using existing labels, the impact of diagnostic labels on actual patient or clinician decisions or the impact of detailed risk information on diagnostic labels, all of which are areas for future research.

## Introduction

 Melanoma incidence and mortality trajectories in Australia and other countries show a classic epidemiological signature of overdiagnosis:^1^ steeply increasing incidence curves coupled with flat mortality trends.^2^,^6^ While ageing populations may lead to a small real increase in melanoma incidence,^7^ much of the increase is likely overdiagnosis.^2^,^6^ This appears to be largely driven by increased diagnosis of melanoma in situ,^2 4 8^ which in Australia is now diagnosed over twice as frequently as invasive melanoma.^9^ Similar findings have been found for melanoma in the USA (diagnosed at least as frequently as invasive melanoma)^3^ and Denmark (diagnosed over half as frequently as invasive melanoma).^10^

Multiple evidence lines indicate that melanoma in situ is a risk factor for invasive melanoma rather than an obligate precursor.^3 9 11 12^ Overdiagnosis is partly driven by lowering the diagnostic threshold over the years, such that the same lesion that was called benign in the past would now be labelled melanoma in situ.^12^ Concerns about litigation may also be driving a tendency to interpret melanocytic lesions as a more severe diagnosis^13^ particularly in partial biopsies or where the lesion extends to the surgical margins. Harms stemming from melanoma overdiagnosis include physical, psychosocial and economic dimensions.^14^ Physical harms can include overtreatment, repeat skin biopsies,^15^ scarring,^15^ pain, infection and/or functional impairment. Psychological harms include anxiety and fear,^16 17^ with many patients perceiving that they have a high risk of dying from melanoma, when their actual risk is much lower (and risk all-cause mortality is actually lower than the population average).^18^ These psychological harms can manifest as anxiety about being outdoors, fear of cancer recurrence, or guilt for past ultraviolet (UV) radiation exposure causing melanoma.^5^ Social harms include impacts of the diagnosis on loved ones, and on patients’ social networks.^15^ Economic harms include treatment costs for the immediate diagnosis and for future long-term clinical surveillance. These incur substantial financial costs to both the health system and patient (as out-of-pocket costs), as well as opportunity costs for both clinician time and patient time. There is also a possible denial of life insurance as the person is now identified as a cancer survivor by many insurance companies.^3^

One possible solution is to consider a new label for melanoma in situ without the word ‘melanoma’.^12^ This might help patients recognise the lower risk of this type of lesion^18^ and help to reduce the potential psychological harm. It may also pave the way for the de-escalation of treatment^19^ and surveillance.^20^,^22^ Evidence from other cancer contexts, including thyroid,^23^ breast^24^ and prostate^25^ lesions, suggests that new diagnostic labels may beneficially impact psychological outcomes and management decisions.^26^ We seek to build on these findings by investigating the potential impacts of new labels for melanoma in situ. To ensure relevance of our findings to end-users, we will test alternative labels for melanoma in situ that were chosen by our co-investigators representing clinicians, patients and the public. Alternative label(s) need to be acceptable to both patients and clinicians, and convey the low, but not zero, risk of future invasive melanoma. This study aims to explore whether using an alternative diagnostic label to communicate a hypothetical melanoma in situ diagnosis influences management choice and level of anxiety among Australian adults.

## Methods and analysis

### Study design

An online randomised study of Australian community members will be run, with participants randomised to receive one of the three hypothetical scenarios about the diagnosis of a melanoma in situ. Each group will be presented with a different diagnostic label, and we will survey participants about their preferred choices of management for that diagnosis, their level of anxiety about that diagnosis and their level of anxiety about their management choices.

This study is a between-subjects randomised online experiment. Following consent, eligible participants will be randomised 1:1:1 to ‘melanoma in situ’ (control), ‘low-risk melanocytic neoplasm’ (intervention label 1) and ‘low-risk melanocytic neoplasm, in situ’ (intervention label 2). The co-primary outcomes and secondary outcomes will be compared across randomised groups.

**Figure 1:**
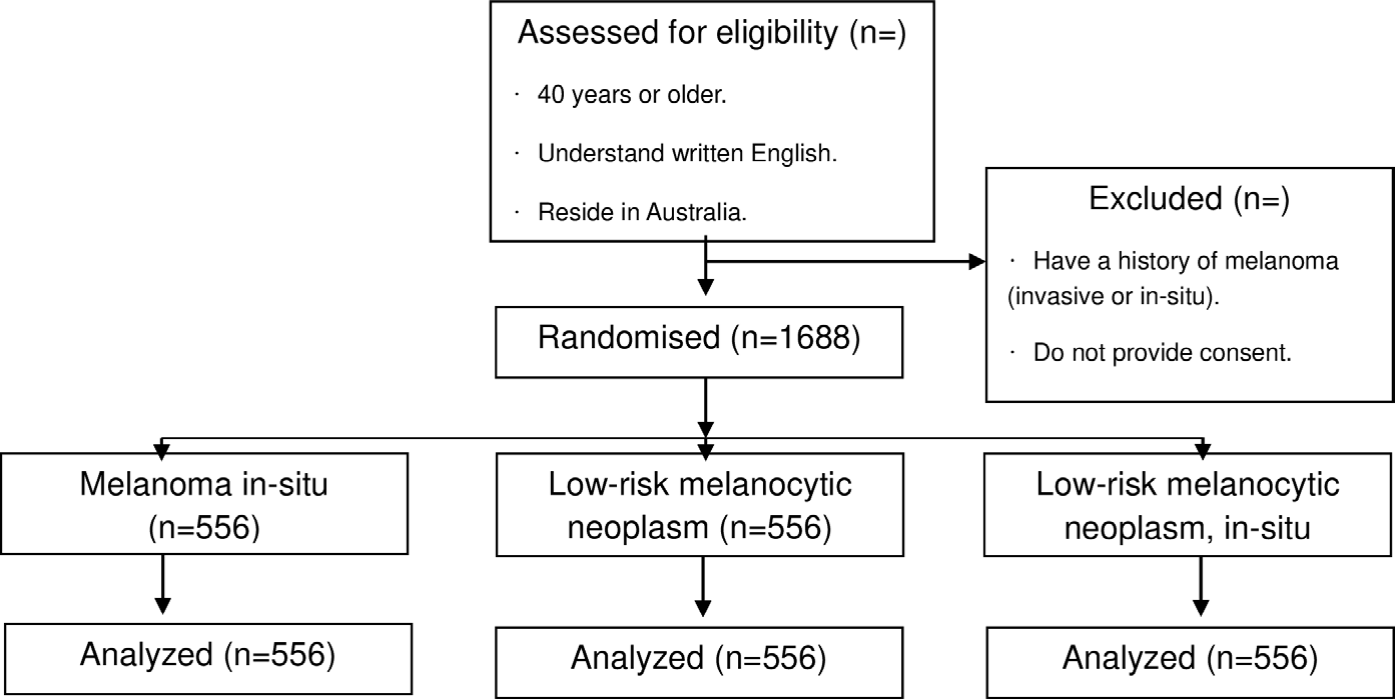
Study Consolidated Standards of Reporting Trials flow diagram for participants. Participants’ selection inclusion criteria are age over 40 years, understanding written English and residing in Australia. Patients will be excluded if they have melanoma or do not provide consent.

There will be an equal probability of being assigned to each of the three groups, and we expect approximately equal numbers per group. We will use Qualtrics survey software to randomly allocate participants into groups, present the scenarios, survey questions and collect data on the outcomes.^27^ Our participants’ flow diagram presents a summary of the randomisation of participants into the allocated control and intervention arms (figure 1).

### Eligibility criteria

Participants will be eligible if they are 40 years or older, understand written English and reside in Australia. Participants will be excluded if they have a history of melanoma (invasive or in situ).

### Recruitment and data collection

Participants will be recruited from the general Australian public through an independent social research company (Dynata), which has a panel of 600 000 participants whose demographic characteristics align closely with those of the national population. Dynata has a point system in which participants receive points after completing surveys. The points can then be used to redeem vouchers, cash or other rewards. Stratified sampling will be used, with quotas in place for gender (50% male, 50% female or other), age (25% for each of 40–29 years, 50–59 years, 60–69 years, 70 years or older, ±15% allowed for first three age groups and ±30% for oldest age group),^28^ education (50% high school or less, 50% more than high school, ±15% allowed) and State or Territory of residence (quotas proportionate to Australian population, ±5% allowed: New South Wales, 31.3%; Victoria, 25.6%; Queensland, 20.5%; South Australia, 6.9%; Western Australia, 10.9%; Tasmania, 2.1%; Northern Territory, 0.9%; and Australian Capital Territory 1.7%).^29^

Participants who agree to participate in the study will complete an online Qualtrics survey managed by the research team. Only eligible participants will proceed to the randomisation step. The survey will capture baseline data and characteristics of participants including socio-demographic details including their age, location, health literacy, and personal and family history of any cancer, and participant responses on outcome measures. The survey questions are presented in the online supplemental file.

All data will be collected via Qualtrics software and hosted on The University of Sydney secure server. Information will be de-identified, and we will not be able to link the survey back to participants. The non-identifiable data will be downloaded for analysis and stored within The University of Sydney’s Research Data Store.

### Determination of alternative labels to be tested

We undertook a targeted literature search in September 2023 by retrieving forward and backward citation searches of four key papers on the topic.^5 12 26 30^ We used the automated tool ‘Spider Cite’^31^ to identify records and Covidence to screen title, abstract and full texts.^32^ Of 593 unique records retrieved, we screened the full text of 27 and included seven papers describing nine alternative labels (see box 1).

Box 1 **Process to select alternative labels to melanoma in situ for testing**

- In the first-round surveys, clinician and patient/public co-investigators indicated their ranking the seven labels identified in the targeted literature search and two additional labels in order of preference. The potential alternative labels from the literature search were as follows: melanocytic neoplasm of low malignant potential (8, 24, 25), melanocytic neoplasm, atypical neoplasm (25), severe or high-grade melanocytic dysplasia, superficial atypical melanocytic proliferation of uncertain malignant significance (SAMPUS) (26–28), melanocytic tumour of uncertain malignant potential (MELTUMP) and melanocytoma (28). The two additional labels suggested by the research team were low-risk melanocytic neoplasm and low-risk melanocytic lesion.
- In the second-round surveys, co-investigators indicated their preferred ranking of the top three choices from round 1 and two new labels suggested in round 1: low-risk melanocytic neoplasm, low-risk melanocytic lesion and melanocytic neoplasm of low malignant potential, melanocytic intraepithelial neoplasia and in situ melanocytic neoplasm.
- In the third-round surveys, co-investigators indicated their preferred ranking of the top two choices from round 2 and three new labels suggested in round 2: in situ melanocytic neoplasm, low-risk melanocytic neoplasm, in situ melanocytic neoplasm, low risk, low-risk melanocytic neoplasm, in situ and dysplastic naevus.

The two highest ranked labels, chosen as the alternative labels to test in the online experiment, were ‘*low-risk melanocytic neoplasm*’ and *‘low-risk melanocytic neoplasm, in-situ*’.

Using short online questionnaires implemented in Qualtrics,^27^ we then ran three rounds of surveys with the nine international clinician co-investigators (with expertise in dermatopathology, dermatology, surgical oncology, primary care and radiation oncology) and six patient/public co-investigators (two with lived experience of a melanoma diagnosis and four without a history of melanoma) to determine choice of alternative labels. This resulted in the final choice of two alternative labels that we will test in the online survey: *low-risk melanocytic neoplasm* and *low-risk melanocytic neoplasm, in situ*.

### Interventions

Participants will be randomised using Qualtrics randomisation software to receive one of the three hypothetical scenarios. They will not be blinded. In each scenario, the participant will be told that the results of their recent skin surgery indicate a particular diagnosis. Group 1 (the control group) will be told they have a *melanoma in situ*. Group 2 will be told that they have a *low-risk melanocytic neoplasm*. Group 3 will be told that they have a *low-risk melanocytic neoplasm, in situ*. We will not provide further explanation of what low risk means.

### Primary and secondary outcomes

Primary and secondary outcomes are described in table 1. The co-primary outcomes are (1) participant’s choice of surgical management option: no further surgery versus further surgery (to achieve pathology margins greater than 5 mm) and (2) follow-up management option: patient-led surveillance (self-skin examination with patient-initiated clinic visits) versus clinician-led surveillance (6 monthly routinely scheduled clinic visits).

**Table 1:** Participant characteristics and outcome measures

Variable	Measure
**Participant characteristics**	
Melanoma risk	Melanoma risk prediction-based self-assessed risk factors^38^
General mood and well-being	WHO (Five) Well-Being Questionnaire^39^
Medical minimiser/maximiser	Single-Item Maximiser/Minimiser Elicitation Question (MM1)^40^
Health literacy	Single Item Literacy Screener (SILS)^41^
Melanoma worry	Direct choice between specified options, one choice possible
Self-efficacy	Generalised Self-Efficacy Scale (GSE)^42^
**Primary outcomes**	
Co-primary outcomes are choices for two management decisions. Choice of further surgery: No further surgery Further surgery to ensure margins>5 mm from lesion on pathology Choice of follow-up: Patient-led surveillance: self-monitoring with patient-initiated clinic visits as needed Clinician-led surveillance: 6 monthly routinely scheduled clinic visits	Direct choice between two management approaches for each co-primary outcomeChoice of further surgery and choice of follow-up
**Secondary outcomes**	
Diagnosis anxiety (feelings)	Single-question Visual Analogue Scale (0–6)^43 44^
Experiential perceived risk (vulnerability)	Single-question Visual Analogue Scale (0–6)^44^
Perceived lifetime absolute risk of invasive melanoma	Single-question Visual Analogue Scale (0–100)^44^
Perceived lifetime comparative risk of invasive melanoma	Single-question Visual Analogue Scale (0–6)^44^
Perceived lifetime risk of dying from melanoma	Single-question Visual Analogue Scale (0–100)
Management choice anxiety	Single-question Visual Analogue Scale (0–6)^43^
Open-text explanation of management choice	Free text (optional)

The first co-primary outcome on surgical management choice reflects recent retrospective analyses that have found that narrower margins are likely to be as safe as margins currently recommended in guidelines in small melanoma in situ.^33^ Indeed, very narrow histological clearance (≥1 mm) appears to be safe for melanoma in situ of the trunk and limbs.^34^ The new MPATH-Dx V2.0 melanocytic lesion classification scheme recommends that provided margins are not involved, and clinicians may consider not re-excising class II lesions—which includes melanoma in situ.^35^ The second co-primary outcome on follow-up management choice centres around patient-led surveillance (also called patient-initiated follow-up) as an alternative model of follow-up for cancer survivors to routinely schedule clinic appointments.^36^ Among people diagnosed and treated for early stage melanoma, patient-led surveillance is being evaluated in the MELanoma SELF surveillance (MEL-SELF) randomised controlled trial. Here, this model of care includes training in self-skin examination, digital technologies to record and take images of concerning lesions (using a mobile dermatoscope), online system for submitting images for remote review by a dermatologist and advice on whether urgent clinical review may be needed (teledermatology).^37^

Secondary outcomes are as follows: diagnosis anxiety, perceived lifetime risk of invasive melanoma, perceived lifetime risk of dying from melanoma, management choice anxiety and open-text explanation of management choices (free text input).

### Sample size

We estimated a sample size of 1668 participants with 556 participants per group in the study, which would provide 80% power (1 - β) to detect a pairwise difference in the proportion of choosing no further surgery and 89% power to detect a pairwise difference in the proportion in choosing patient-led surveillance as small as 10%.

The assumptions are 50% would choose no further surgery (most conservative assumption) and 35%^22^ would choose patient-led surveillance in the control label condition, a 5% dropout rate, α=0.05, the normal approximation to the binomial distribution and the standard formula for comparing proportions in independent equal-sized groups.

### Analysis

The analysis will focus on assessing the impact of different diagnostic labels for melanoma in situ on participants’ psychological responses and healthcare decisions. Data analysts will be blinded to intervention assignment. For both co-primary outcomes, we will compare the proportion chosen for each management option. For first four secondary outcomes, we will compare summary statistical measures (means or medians) across randomised groups. For the last outcome, we will use thematic framework methods of qualitative data.

The analysis will adhere to the intention-to-treat principle, and participant data will be analysed according to their randomly assigned diagnostic label group, regardless of adherence to the study protocol. The number of participant responses included in each analysis will be presented for each outcome. We will summarise categorical data for the randomised groups using counts and percentages, and continuous data using the minimum and maximum, mean and SD or median and IQR.

Statistical analyses will be conducted within a superiority framework to make pairwise comparisons across the three diagnostic label groups. Binary outcomes will be analysed using logistic regression. Continuous outcomes will be analysed using linear regression. For the cancer worry outcome, we will compare changes in worry across randomised groups by including baseline scores as a covariate in the regression model. Effect estimates for all primary and secondary outcomes will be presented with associated 95% CI. All hypothesis tests will be two-sided with a significance level (α) of 5%. The potential for participants’ health literacy to act as an effect modifier of intervention effects will be explored.

We will estimate unadjusted and adjusted effects using the relevant regression model. These will include variables used in sampling strata: age, education and geographic location (by state/territory). Prognostic factors will be measured through the baseline questionnaire and include baseline anxiety levels, sun exposure behaviour, prior diagnosis of melanoma and diagnosis of melanoma in a family member. The effects of participants’ health literacy on intervention effects will also be explored as a potential confounder.

### Planned start and end dates for the study

The anticipated date of first participant enrolment was 01 July 2024, and the anticipated date of last data collection completion was 01 August 2024 (see Australian New Zealand Clinical Trials Registry, ID: ACTRN12624000740594).

### Patient and public involvement

Two authors have lived experience of a melanoma diagnosis (one had MIS and one had a thin stage I invasive melanoma), and four authors are members of the public. Two authors are affiliated with Cancer Voices New South Wales (NSW), one author is a patient researcher from Cambridge UK, and three authors are affiliated with Health Consumers NSW.

## Ethics and dissemination

Ethics approval of this project was provided by The University of Sydney on 06 May 2024 (No. 2024/HE000019). The study is registered with the Australian New Zealand Clinical Trials Registry (ACTRN12624000740594). Updates to the protocol will be uploaded to the registry and identified by version number.

As this study is an online randomised experiment which includes a hypothetical scenario, we do not anticipate significant adverse events because of the trial interventions or conduct. Participants are reminded at several points before and after the study as part of the participant information, consent and debrief processes that the nature of the study is hypothetical, that none of the information relates to their actual health or well-being and that researchers do not have access to their actual medical histories or information. The debriefing content also includes links to relevant resources for participants who wish to find out more.

### Data availability statement

The research team will have access to the final dataset. Access may be granted to other researchers on reasonable request. No contractual agreements limit the disclosure of data to other investigators. The findings of the study will be published in a peer-reviewed medical journal. A lay summary of the findings will be published via permanent link at the Wiser Healthcare publications page.

## supplementary material

10.1136/bmjopen-2024-089558 online supplemental file 1
