# Supplementary material for: Impact of alternative diagnostic labels for melanoma in situ on management choices and psychological outcomes: protocol for an online randomised study
Source: BMJ Open. 2024 Dec 20;14(12):e089558. doi: 10.1136/bmjopen-2024-089558 (PMC11667480; doi:10.1136/bmjopen-2024-089558)
Supplement: online supplemental file 1 [file bmjopen-14-12-s001.pdf]

Landing Page ✓

Does the label for a low-risk melanocytic lesion influence management choice: a randomised experiment.

Thank you for your interest in our study about low-risk melanocytic lesions.

In this study, you will be randomised to be shown one of three hypothetical scenarios following surgery on a mole, which will be followed by questions about management options and anxiety.

The study is being conducted by a team of researchers from The University of Sydney School of Public Health. The team members are:

- Professor Katy Bell (School of Public Health at the University of Sydney)
- Dr Brooke Nickel (School of Public Health at the University of Sydney)
- Mr Zhuohan Wu (School of Public Health at the University of Sydney)

Taking part in the study involves completing one online questionnaire which will take approximately 10 minutes to complete.

Being in this study is completely voluntary and you do not have to take part. Your decision on whether to participate will not affect your current or future relationship with the researchers or anyone else at the University of Sydney.

Please take the time to read through the Participant Information Statement below.

If you are interested in taking part in this study, you will be asked to consent to take part by ticking the 'yes' box at the beginning of the questionnaire. By giving your consent to take part in this study, you are telling us that you:

✓ Understand what you have read in the Participant Information Statement.
✓ Agree to take part in the research study as outlined in Participant Information Statement.
✓ Agree to the use of your personal information as described.

When you have consented, you will fill out an online questionnaire that asks a series of questions, such as:

- Demographic questions, such as age, education, income level and relationship status.
- General health and cancer related questions.
- Melanoma and other cancer history related questions.

You will be randomised to read one of three **HYPOTHETICAL EXAMPLES** (these are made-up examples) in which different labels are used to explain a low-risk melanocytic skin lesion result. **Please note that you WILL NOT be receiving information or advice on any real mole check results or information about your actual health status**.

The hypothetical examples will be followed by questions about choice of management strategy and personal perspective.

Note that there is no back button. Please give your best answer to each question before moving on to the next.

Pre-Survey PIS ✓

You can click the link below to download the Participants Information Sheet for more information about this study.

Participants Information Sheet

Pre-Survey Consent Form ✓

Do you consent to take part in this study as described in the welcome page and Participants Information Sheet?

- ◯ Yes
- ◯ No

Section 1: Screening and Socio-Demographic ✓

Have you been previously diagnosed with a melanoma?

- ◯ Yes
- ◯ No

What is your age?

Section 1.5: Screening and Socio-Demographic Part 2

Which of the following best describes your current gender identify?

- Male
- Female
- Non-binary / gender fluid
- Different identify

Which Australian state or territory do you currently live in?

- New South Wales
- Victoria
- Australian Capital Territory
- Queensland
- South Australia
- Western Australia
- Northern Territory
- Tasmania

Where are you located? (please enter your post code)

What is your highest level of education?

- Year 10 or below
- Year 11
- Year 12
- Certificate I/II
- Certfficate III/IV
- Advanced diploma/diploma
- Bachelor's degree
- Graduate diploma/graduate certificate
- Postgraduate degree (Masters or Doctorate)

- Other - please specify: ____

What is your current employment status?

- Permanent or ongoing
- Fixed-term contract
- Casual/temporary (no paid sick leave or annual leave)
- Self-employed
- On paid leave (e.g. maternity leave)
- Unemployed
- Not working/not in the labour force (e.g. student, home duties, retired)

What was your total household income before taxes during the past 12 months?

- Less than AUD $30,000
- Between AUD $30,000 - $49,999
- Between AUD $50,000 - $79,999
- Between AUD $80,000 - $99,999
- Between AUD $100,000 - $149,999
- Between AUD $150,000 - $199,999
- AUD $200,000 or more
- Prefer not to say

Do you have private health insurance?

- Yes
- No
- Don't know

Do you have a partner?

- Spouse
- De-facto partner
- Partner who does not reside with you
- No partner
- Widowed
- Divorced or separated
- ______ Other - please list:

Do you have children?

- Yes
- No
- Prefer not to say

Are you of Aboriginal or Torres Strait Islander origin?

- Aboriginal
- Torres Strait Islander
- Both Aboriginal and Torres Strait Islander
- Neither Aboriginal or Torres Strait Islander
- Prefer not to say

Were you born in Australia?

- Yes
- No

What is your country of birth?

- ◯ UK
- ◯ India
- ◯ China
- ◯ New Zealand
- ◯ The Philippines
- ◯ [ ] Other - please list:

In which year did you move to Australia?

[ ]

What language do you mostly speak at home?

- ◯ English
- ◯ Mandarin
- ◯ Arabic
- ◯ Cantonese
- ◯ Vietnamese
- ◯ [ ] Other - please list:

What was your natural hair colour when you were 18 years of age

- ◯ Black
- ◯ Brown
- ◯ Fair or Blond
- ◯ Red or Auburn

Looking at the image below, please select the option that approximately represents the number of moles on your body when you were aged 18 years, as best as you can remember.

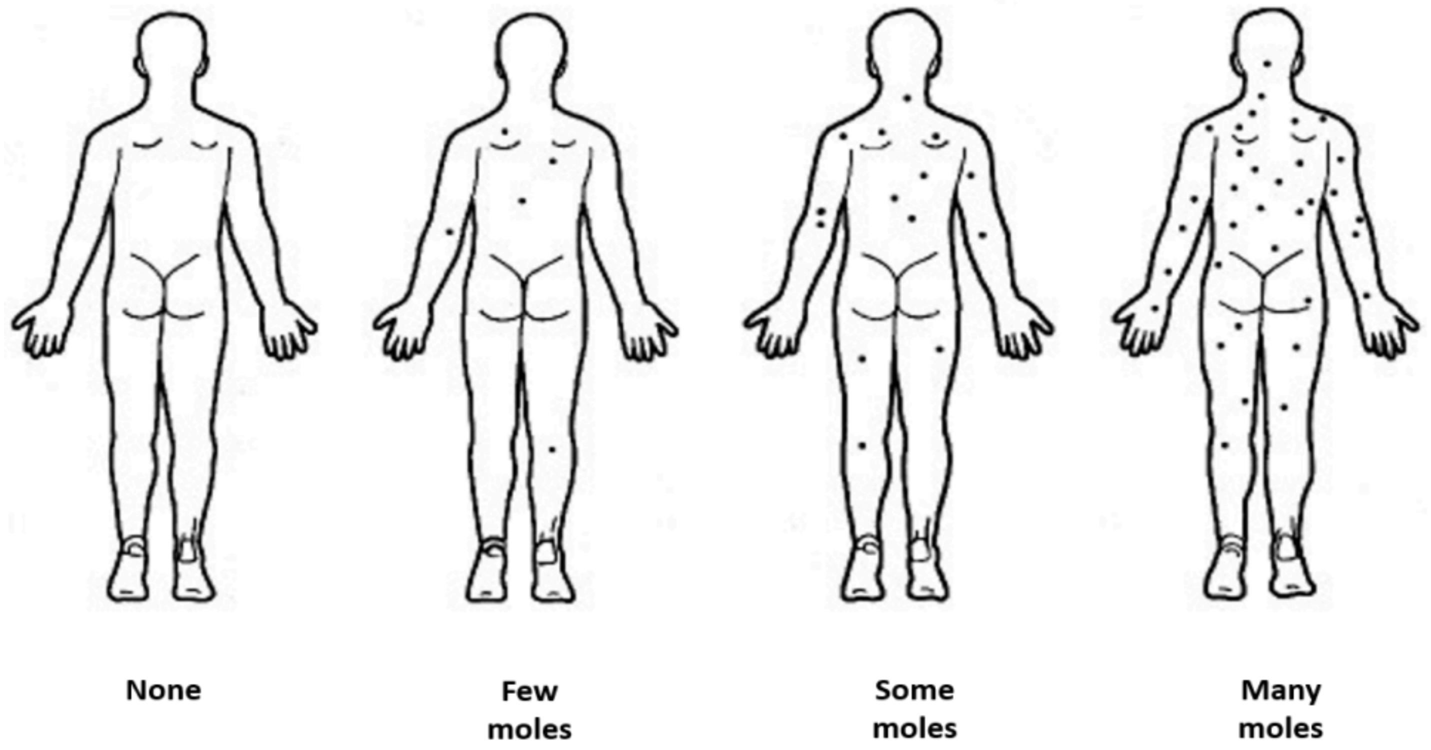

- ◯ None
- ◯ Few moles
- ◯ Some moles
- ◯ Many moles

Have you ever used a sunbed or sunlamp?

- ◯ Yes
- ◯ No

Section 2: General Health Screening

Have you ever been diagnosed with any type of cancer?

- ◯ Yes
- ◯ No
- ◯ Don't know

Which type of cancer?

- ☐ Melanoma
- ☐ Skin (not melanoma)
- ☐ Prostate
- ☐ Breast
- ☐ Bowel
- ☐ Lung
- ☐ Lymphoma
- ☐ ______ Other - please list:
- ☐ Don't know

Section 2: General Health ✓

In general, would you say your health is ...

- ◯ Excellent
- ◯ Very good
- ◯ Good
- ◯ Fair
- ◯ Poor

Has a current or former partner or a close friend ever been diagnosed with cancer?

- ◯ Yes

- No

Which type of cancer?

- Melanoma
- Skin (not melanoma)
- Prostate
- Breast
- Bowel
- Lung
- Lymphoma
- Other - please list:
- Don't know

Has anyone in your close family ever been diagnosed with cancer?

- Yes
- No
- Don't know

Which type of cancer? Please tick all that apply

- Melanoma
- Skin (not melanoma)
- Prostate
- Breast
- Bowel
- Lung
- Lymphoma
- Other - please list:

- ☐ Don't know

Who was this? Please tick all that apply

- ○ Mother
- ○ Father
- ○ Sister
- ○ Brother
- ○ Daughter
- ○ Son
- ○ ______ Other - please list:

How worried are you about developing melanoma?

- ○ Not worried at all
- ○ A bit worried
- ○ Quite worried
- ○ Very worried

Sometimes, medical action is clearly necessary and sometimes it is clearly not necessary.

Other times, reasonable people differ in their beliefs about whether medical action is needed.

In situations where it’s not clear, do you tend to lean towards taking action or do you prefer to wait and see if action is needed?

Importantly, there is no right way to be.

- ○ I strongly lean towards wait and see.

◯ I lean towards wait and see.
◯ I somewhat lean towards wait and see.
◯ I somewhat lean towards taking action.
◯ I lean towards taking action.
◯ I strongly lean towards taking action.

The following questions are related to how you have been feeling over the past two weeks. Please read each statement and then choose the most appropriate option regarding how you felt in the last two weeks.

	At no time	Some of the time	Less than half of the time	More than half of the time	Most of the time	All of the time
I have felt cheerful and in good spirits.	◯	◯	◯	◯	◯	◯
I have felt calm and relaxed.	◯	◯	◯	◯	◯	◯
I have felt active and vigorous.	◯	◯	◯	◯	◯	◯
I woke up feeling fresh and rested.	◯	◯	◯	◯	◯	◯
My daily life has been filled with things that interest me.	◯	◯	◯	◯	◯	◯

Please respond to the following statements.

	Not at all true	Hardly true	Moderately true	Mostly true
I can always manage to solve difficult problems if I try hard enough.	◯	◯	◯	◯
If someone opposes me, I can find the means and	◯	◯	◯	◯

	Not at all true	Hardly true	Moderately true	Mostly true
ways to get what I want.				
It is easy for me to stick to my aims and accomplish my goals.	◯	◯	◯	◯
I am confident that I could deal efficiently with unexpected events.	◯	◯	◯	◯
Thanks to my resourcefulness, I know how to handle unforeseen situations.	◯	◯	◯	◯

	Not at all true	Hardly true	Moderately true	Mostly true
I can solve most problems if I invest the necessary effort.	◯	◯	◯	◯
I can remain calm when facing difficulties because I can rely on my coping abilities.	◯	◯	◯	◯
When I am confronted with a problem, I can usually find several solutions.	◯	◯	◯	◯
If I am in trouble, I can usually think of a solution.	◯	◯	◯	◯
I can usually handle whatever comes my way.	◯	◯	◯	◯

Section 3: Health Literacy ✓

How often do you need to have someone help you when you read instructions, pamphlets or other written material from your doctor or pharmacy?

◯ Always

◯ Often

◯ Sometimes

◯ Occasionally

◯ Never

Randomized hypothetical labels

Please read the hypothetical information below and answer the questions that follow. You are asked to imagine as if the following information is true. Please answer how you would feel or react if you were in this situation, to the best of your ability.

You are at the doctor (GP) after you recently had a small surgery done to remove one of your moles.

The doctor has the pathology test results and says: “We found a **${e://Field/Label}**. We removed it all, and also 3mm of normal skin around the **${e://Field/Label}**."

Section 5: Primary and Secondary Outcome Measures ✓

Given the diagnosis of ${e://Field/Label}, how anxious do you feel?
Answer from Not at all anxious (0) to Extremely anxious(6).

	Not at all				Extremely	
0	1	2	3	4	5	6

Given the diagnosis of ${e://Field/Label}, how vulnerable do you feel to developing invasive melanoma sometime in your life?
Answer from Not at all vulnerable (0) to Extremely vulnerable (6).

Not at all				Extremely		
0	1	2	3	4	5	6

Given the diagnosis of ${e://Field/Label}, on a scale of 0–100%, what do you think your chances are of **developing** an invasive melanoma sometime in your life?

		Low risk (%)					High risk (%)			
0	10	20	30	40	50	60	70	80	90	100

Given the diagnosis of ${e://Field/Label}, what do you think your chances are of developing an invasive melanoma, compared to others of your age, gender, and skin colour?
Answer from Much lower chance (0) to Much higher chance (6).

Much lower				Much higher		
0	1	2	3	4	5	6

Given the diagnosis of ${e://Field/Label}, on a scale of 0 –100%, what do you think your chances are of **dying** from melanoma?

Low risk (%)							High risk (%)			
0	10	20	30	40	50	60	70	80	90	100

Please explain your reasoning behind the percentage you provided. [This question is optional]

Your doctor continues: "You now need to decide whether you would like us to do further surgery to remove more normal skin from around the scar, or whether you would prefer no further surgery at this time. I recommend either of these options as a reasonable choice and will organise whichever you prefer."

Given the diagnosis of ${e://Field/Label}, which of these surgery management options would you choose?

- Further surgery to remove more normal skin around the scar (so that the distance from the margins to the **${e://Field/Label}** is greater than 5 mm)
- No further surgery

Please tell us how you decided on that surgery management option. What were the important factors that helped you decide? [This question is optional].

After making that surgery management choice, how anxious do you feel?
Answer from Not at all anxious (0) to Extremely anxious (6).

Not at all				Extremely		
0	1	2	3	4	5	6

Your doctor further explains that there are also different options for follow-up: "You need to also decide whether you would like to book in for regular skin checks with me every 6 months, or you would like us to teach you how to check your skin yourself (with tele-dermatologist support) and book in with me only if needed. Again, I recommend either option as a reasonable choice, and will organise whichever one you prefer."

Given the diagnosis of ${e://Field/Label}, which of these follow-up management options would you choose?

- ◯ My doctor does my skin check at regular 6 monthly appointments.
- ◯ I do my own skin checks with help from my partner/friend/relative (to check my back and other hard to see areas), and book in with my doctor when I need to.
    - I am taught how to examine my total body and am given a special imaging device that clips on my phone.
    - I have access to videos and online support to help me do skin checks and use the imaging device.
    - I can take images of any moles that concern me and send these to a dermatologist**.**
    - If the dermatologist is concerned, them I am booked in immediately for a skin check with my doctor.

Please tell us how you decided on that follow up management option. What were the important factors that helped you decide? [This question is optional].

After making that follow up management choice, how anxious do you feel? *Answer from Not at all anxious (0) to Extremely anxious (6).*

Not at all				Extremely		
0	1	2	3	4	5	6

Section 6: Debrief Statement ✓

You were a participant in this study which aimed to investigate how people would react to different information provision on diagnosis of a low-risk melanocytic lesion results by the label given to the melanocytic lesion.

During the study, you were asked to imagine a hypothetical scenario in which you are given a diagnosis result after having gone to a routine screening. You

were then asked to complete a series of survey questions.

You were randomised to receive one of three different hypothetical scenarios.

These three diagnosis scenarios were:

1. Diagnosis of a melanoma in situ.
2. Diagnosis of a low-risk melanocytic neoplasm.
3. Diagnosis of a low-risk melanocytic neoplasm, in situ.

The purpose of this study was to examine the impact of these different labels/diagnoses on preferred management strategy and psychological outcomes such as worry and health seeking intentions.

It is important to remember that this study was entirely hypothetical (made up). The study team does not have access to any of your medical history.

If you have any further questions regarding the study, feel free to contact Prof Katy Bell (katy.bell@sydney.edu.au)

For more information on melanoma and skin checks, please visit the following websites:

Melanoma Institute Australia
Cancer Council - Melanoma

We are conducting a follow-up study to explore individuals' experiences, concerns, and preferences regarding current and potential alternative labels for melanoma in situ. Would you be interested in participating in an interview

over the telephone or using web conferencing tools like Zoom or Microsoft Teams?

- ◯ Yes
- ◯ No

Thank you for your interest! Please provide your email address below so we can contact you to schedule the interview.

Section 7: Feedback ✓

Thank you for your participation in the survey. Your time and contribution is greatly appreciated. If you are interested in the results of the study, the results and a lay summary of the results will be published at the following permanent web page: Wiser Healthcare publications

Powered by Qualtrics
