# Supplementary material for: Impact of alternative diagnostic labels for melanoma in situ on management choices and psychological outcomes: protocol for an online randomised study
Source: BMJ Open. 2024 Dec 20;14(12):e089558. doi: 10.1136/bmjopen-2024-089558 (PMC11667480; doi:10.1136/bmjopen-2024-089558)
Supplement: Supplementary file 2 [file bmjopen-2024-089558.reviewer_comments.pdf]

PEER REVIEW HISTORY

BMJ Open publishes all reviews undertaken for accepted manuscripts. Reviewers are asked to complete a checklist review form and are provided with free text boxes to elaborate on their assessment. These free text comments are reproduced below.

ARTICLE DETAILS

Title (Provisional)

Impact of alternative diagnostic labels for melanoma in-situ on management choices and psychological outcomes: protocol for an online randomised study.

Authors

Wu, Zhuohan; Nickel, Brooke; boroumand, farzaneh; Elder, David; Ferguson, Peter M.; Scolyer, Richard Anthony; O'Brien, Blake; Barnhill, Raymond; Adamson, Adewole S.; van Akkooi, Alexander C.J.; Emery, Jon; Parker, Lisa; Low, Donald; Low, Cynthia; Davies, Elspeth; Liu, Sherrie; Lewis, Stacey; Spongberg-Ross, Bella; Bell, Katy

VERSION 1 - REVIEW

**Reviewer**	**1**
**Name**	**Swerlick, Robert A.**
**Affiliation**	**Emory University**
**Date**	**12-Aug-2024**
**COI**	**None**

This manuscript describes a proposed study to examine the effects of a proposed change in terminology for ambiguous pigmented lesions and possible effects on inform patient decision making. This is an important study which may ultimately substantially influence how pigmented lesions of the skin are managed.

The investigators have already done preliminary work and have identified suitable language to test. They have decided to focus on melanoma in situ and the alternative nomenclatures low risk melanocytic neoplasm, and low risk melanocytic neoplasm, in situ. The melanoma in situ term is already widely used and represents the current nomenclature. Whether or not low risk melanocytic neoplasm (with or without in situ) can or will be embraced in the future it is not entirely relevant to the research question at hand and I believe these terms will suffice within this study.

The consort flow diagram is clear with the exception of age exclusions. In the text, the investigators note a 40 and over year cutoff while in the consort diagram it is listed as 18 years or older. This should be clarified. The investigators will randomize 1688 subjects with 556 in each of the different nomenclatures. I am not in a position to confirm the power calculations will be sufficient to detect differences but the numbers seem plausible.

The investigators that the potential for participants health literacy to act as a modifier of intervention effects will be explored. Similarly they note the effects of the participants health

literacy on intervention effects will be explored is a potential confounder. This is a major issue and it would be ideal if this can be addressed at randomization.

The investigators query subjects as to whether they tend to lean towards action or not and one of the questions. I believe this is an extremely important tendency to capture among their subjects. I believe the study would also be enhanced by adding a question or questions relating to subject numeracy.

I have a major problem with questions relating to risk of developing an invasive melanoma or risk of dying from melanoma that are posed to subjects. Presentation of risk as percentages may not be optimal and it may be more desirable to present risk in terms of absolute frequencies or relative frequencies. In addition the scales used may not be relevant to the questions posed. For example a high lifetime risk of melanoma death for thin melanomas may be viewed is no higher than 25 to 30%. However on the scales presented to the subjects a 30% lifetime risk of death if shown is low risk.

The referencing of the manuscript is incomplete. The reference list attached extends to reference 26. However, in the tables, references continue on to reference 31.

**Reviewer**	**2**
**Name**	**Muir, James**
**Affiliation**	**The University of Queensland**
**Date**	**19-Sep-2024**
**COI**	**I have no competing interests.**

This is a topic close to my heart. Especially since the (incredible) decision as made to increase excision margins for MIS to 10 mm. So I have written a bit of a tome.

In summary 'good idea but has flaws and could be a lot better'.

When I read the article lots of questions sprang to mind. some were answered in the questionaire reading. You need to address these in the article. For example your idea of 'self examination' is not communicated very well in the article. In the hypothetical they are taught self exam. In the primary and secondary outcomes measures you offer them a very sophisticated telemedicine service. Not widely available. This is confusing.

In the abstract you state " but existing terminology makes it difficult for patients to

distinguish these from higher risk invasive melanomas." This is true but as importantly does this not apply to doctors? The clinicians enrol patients in short term digital dermoscopic imaging trying to capture these lesions, they get histopath reviewed if the report does not read MIS, the pathologists order an array of extra stains and come up with diagnoses such as 'best regarded as MIS" or "evolving MIS" etc, Clinicians then follow the guidelines and do 10 mm margins where they used to do 5 or when the histopath report reads '3 mm clear histological margin' they re-operate to get 5 mm. So I think in fairness you need to discuss this aspect (I think your study is a great idea and reflects local practice here. We tell them 'this lesion is harmless, the trip here for a skin check is more dangerous.).

Will you give them an idea of what 'low risk" means?

As the study group has no 'skin in the game' i.e. they are not MIS patients will your results help us much?

I wonder what a treating clinician would do if called upon to treat a patient way outside of guidelines . where I work the melanoma guidelines are regarded as Holy Writ by some.

Where did 0.5 mm come from as an adequate margin? Why not 1 or 2 mm, "clear" or 'well clear"? you should explain that.

Also how much greater than 0.5 mm was the re excision to achieve? Do you mean 'usual treatment as per the guidelines'? If so might be better to state that.

Will the choice of patient led follow up be accompanied by education in same? (the evidence re skin self surveillance is pretty damning in the main after all).

You state "The randomised design will provide highly relevant evidence on potential

impacts of alternative diagnostic labels for melanoma in situ to patients'

decision-making and psychological outcomes." Is this true if they do not have 'skin in the game'?

also don't we need to know if there is a significant body of evidence that a 0.5 mm histological margin is safe? This would likely be true for smaller MIS but would it be true for a largish lentigo maligna? If I was a member of the public told they had an MIS I would immediately google it. This will tell us to do a wide excision. So that simple search will influence your subjects in the real world.

Won't your survey participants be googling the topic? If some do and some do not that might influence their answers?

"One possible solution is to consider a new label for melanoma in situ without the

word "melanoma". This might help patients recognize the lower risk of this type of

lesion, and help to reduce the potential psychological harm. It may also pave the

way for the de-escalation of treatment and surveillance."

I agree with your introduction. I work in a region with the highest incidence of melanoma in the world. MIS (real or imagined) abound. Doctors are a large part of this issue. Very common for the patient to never have been told (or at least not know) what their diagnosis of MIS actually means. Nor do they understand why a wide excision is advised i.e. life saving v reduced local recurrence. For this study to have greater relevance why not survey doctors involved in MIS management and see what they would do? after all as it stands very few clinicians will go outside guidelines and actually tell a patient they have the alternative diagnostic label and management plan. I recognise you surveyed clinician coinvestigators but what will the great mass of doctors looking after MIS make of these labels/management options? In australia this is largely done by GPs. Dermatopathologists were surveyed but they are not called upon to make actual management decisions in MIS. Very few MIS end up in the hands of surgical or radiation oncologists.I think to have more veritas you would want to know what GPs/Dermos/Plastics/General surgeons think. Interesting to know and will improve the impact of your paper.

You even state "Alternative label(s) need to be acceptable to both patients and clinicians, and convey the low, but not zero, risk of future invasive melanoma." To me this study is of interest but nobody would use it to inform practice. That is fine of course as this is an important discussion. Management decisions are a 'consensus' between patient and clinician. so expanding your survey to clinicians will add to the impact. Easily done.

"patients will be excluded if they have a history of melanoma.' I understand the rationale but it detracts from your study. You include people "40 years or older, understand written English, and reside in Australia." So what if you have a representation of people at very low risk of melanoma i.e. dark skinned ? Will they give the same weight to the hypothetical as red heads with sun damage? What about a patient who has never had melanoma but has a strong family history of same? Or one who has had keratinocyte cancer in the past etc? What about patients who are already 'skin aware' and getting checks despite having no risk factors (heaps of them out there). the point being you are excluding melanoma patients so why not these groups? I would have thought people who have had an MIS would be of equal interest as people at high risk of same?

In the survey will the terms ' melanocytic', 'neoplasm', 'low risk', 'in situ' be defined? Your participants will likely 'google' these. Might effect their answers if they do?

If i was designing this I would confine it to people who have a real/perceived risk of developing melanoma. I recognise you will be collecting this data but would seem logical to survey people with 'skin in the game'.

"Secondary outcomes are: diagnosis anxiety, perceived lifetime risk of invasive melanoma, perceived lifetime risk of

dying from melanoma, management choice anxiety, and open-text explanation of management choices (free text input)." They will google this won't they? In other words will they make the assesment based on the information the question provides or do what people do in ral life? Either you need to prevent them from doing this in some way (makes it articficial) or ask the question 'did you do your own research'?

"The analysis will focus on assessing the impact of different diagnostic labels for melanoma in situ on participants' psychological responses and healthcare decisions." This is a bit artificial (I recognise it has to be). A patient will generally make the decision the doctor tells them to make. With your three options the major determinant ill be what their clinician thinks. The commonest thing we hear when presenting management options is 'what would you do?'. The next is 'I will do what you tell me to do.'

Maybe a better way to field the choices is for them to read an advice from the doctor which says 'You have LABEL HERE. In this situation my advice is WIDE EXCISION/NO FURTHER TREATMENT. YOUR FOLLOW UP SHOULD BE. xyz.. YOUR OTHER OPTIONS ARE A or b. I would consider them reasonable but they have these disadvantages....."

In the hypothetical the doctor leaves it up to the patient to pick an option with the statement 'I reccommend any of these options as reasonable'.. Is that realistic? I think in the main doctors have pretty strong views on what is the 'right way forward' and equally patients come to us for specific advice.

You do not mention the 0.5 mm histology clearance in the hypothetical as far as I can see?

After all no patient makes such an important decision based on the diagnosis alone. they always seek more information and guidance, do their own research etc.

"Two authors have lived experience of a melanoma diagnosis" Were these of MIS only? That is likely relevant.

Can you make this claim from this study? "The potential significance of this study lies in its ability to impact clinical practice and policy by identifying alternative diagnostic labels for melanoma in-situ that are acceptable to patients, pathologists, and treating clinicians." You are only surveying members of the public. We can make no inferences as to what pathologists and clinicians think of these without surveying them can we?

"Alternative labeling of melanoma in-situ may better inform patients about the low level of risk....." The big thing is likely not the label but the management. We tell patients 'you have had an MIS. The lesion is all out. the risk from this is minimal, in fact recent studies have shown that your chances of being alive in 5 years are higher than those who have not had a MIS. However we need to take an extra 10 mm margin." After all with a BCC, IEC, low risk SCC, KA we just treat it and say 'that is it no further treatment needed.' similar issue i.e. a negligible risk lesion but a very different management paradigm.

In the article you state the study is restricted to 40 years and older. In your table it states 18 and older to be legible.

In the article you talk about 'self examination' but in the questionaire it turns out you are going to get them involved in a telemedicine service. You need to make it clear in the article what you mean by self examination , this is not what most people would consider self examination is it?

For me this is a good idea but could be a lot better. It will not inform future practice but it will support/inform further research into changing the management of MIS. I think merely knowing what a theoretical patient thinks is not nearly as useful as what their treating doctor would also think.

Any observer of the clinical 'scene' will recognise that there is a large body of clinicians and pathologists who agonise over the diagnosis of MIS. We regularly see reports where the pathologist has sought multiple opinions, ordered an array of stains etc to distinguish 'severe atypia/high grade dysplasia' from MIS. Often pathologists will not give a firm diagnosis but say 'on balance best regraded as MIS'. Casual observers of the dermoscopy web sites will see regular tales of clinicians demanding more opinions/deepers until a 'correct' diagnosis of MIS is rendered.

So I would suggest you survey all involved in MIS (patients, clinicians and pathologists) re this. Preferably as part of the study or in future.

The article is a bit confusing/misleading at times, see above.

VERSION 1 - AUTHOR RESPONSE

Reviewer 1

1. This manuscript describes a proposed study to examine the effects of a proposed change in terminology for ambiguous pigmented lesions and possible effects on inform patient decision making. This is an important study which may ultimately substantially influence how pigmented lesions of the skin are managed.
The investigators have already done preliminary work and have identified suitable language to test. They have decided to focus on melanoma in situ and the alternative nomenclatures low risk melanocytic neoplasm, and low risk melanocytic neoplasm, in situ. The melanoma in situ term is already widely used and represents the current nomenclature. Whether or not low risk melanocytic neoplasm (with or without in situ) can or will be embraced in the future it is not entirely relevant to the research question at hand and I believe these terms will suffice within this study.

Response: Thank you for your supportive comments. By choosing labels that were acceptable to clinician and patient co-authors we hope to present alternative labels that could potentially be embraced.

2. The consort flow diagram is clear with the exception of age exclusions. In the text, the investigators note a 40 and over year cutoff while in the consort diagram it is listed as 18 years or older. This should be clarified. The investigators will randomize 1688 subjects with 556 in each of the different nomenclatures. I am not in a position to confirm the power calculations will be sufficient to detect differences but the numbers seem plausible.

Response and Change: The eligible age has been corrected to 40 and over in the diagram.

3. The investigators that the potential for participants health literacy to act as a modifier of intervention effects will be explored. Similarly they note the effects of the participants health literacy on intervention effects will be explored is a potential confounder. This is a major issue and it would be ideal if this can be addressed at randomization.

Response: We included education as one of the sampling strata (see response to Editors' comment #5 above). We anticipate that amount of education and health literacy will be balanced across the randomised conditions.

4. The investigators query subjects as to whether they tend to lean towards action or not and one of the questions. I believe this is an extremely important tendency to capture among their subjects. I believe the study would also be enhanced by adding a question or questions relating to subject numeracy.

Response: We agree that this may have been useful to include, however as the data collection is now complete we cannot add further questions.

5. I have a major problem with questions relating to risk of developing an invasive melanoma or risk of dying from melanoma that are posed to subjects. Presentation of risk as percentages may not be optimal and it may be more desirable to present risk in terms of absolute frequencies or relative frequencies. In addition the scales used may not be relevant to the questions posed. For example a high lifetime risk of melanoma death for thin melanomas may be viewed is no higher than 25 to 30%. However on the scales presented to the subjects a 30% lifetime risk of death if shown is low risk.

Response: Thank you for raising this important point. We agree that many people have difficulty in interpreting risk. We now include questions to assess perceived risk of future invasive melanoma using experiential (vulnerability), affective (feelings), and cognitive (absolute and relative numerical measures).

Change (Table 1 pages 29-30, new risk perception measures added as Secondary Outcomes):

Table 1. Participant characteristics and outcome measures.

Variable	Measure
Participant Characteristics	
Melanoma risk	Melanoma risk prediction based self-assessed risk factors[39]
General mood and wellbeing.	WHO (Five) Well-Being Questionnaire.[40]
Medical minimiser/maximiser.	Single-Item Maximiser/Minimiser Elicitation Question (MM1).[41]
Health literacy.	Single Item Literacy Screener (SILS).[42]
Melanoma worry.	Direct choice between specified options, one choice possible.
Self-efficacy.	Generalized Self-Efficacy Scale (GSE).[43]
Primary Outcomes	
Co-primary outcomes are choices for two management decisions.<br>1. Choice of further surgery:<br>• No further surgery<br>• Further surgery to ensure margins >0.5mm from lesion on pathology<br>2. Choice of follow-up:<br>• Patient led surveillance: self-monitoring with patient-initiated clinic visits as needed<br>• Clinician led surveillance: six monthly routinely scheduled clinic visits	Direct choice between two management approaches for each co-primary outcome Choice of further surgery and choice of follow-up.
Secondary Outcomes	
Diagnosis anxiety (feelings)	Single-question Visual Analogue Scale (0-6).[44 45]
Experiential perceived risk (vulnerability)	Single-question Visual Analogue Scale (0-6).[45]
Perceived lifetime absolute risk of invasive melanoma	Single-question Visual Analogue Scale (0-100).[45]
Perceived lifetime comparative risk of invasive melanoma	Single-question Visual Analogue Scale (0-6).[45]
Perceived lifetime risk of dying from melanoma	Single-question Visual Analogue Scale (0-100).
Management choice anxiety.	Single-question Visual Analogue Scale (0-6).[44]
Open-text explanation of management choice.	Free text (optional)

Change (Supplement, page 14-15): Questions on perceived risk:

Affective measure:

Given the diagnosis of (given label), how anxious do you feel? Answer from Not at all anxious (0) to Extremely anxious (6).

Experiential measure:

Given the diagnosis of (given label), how vulnerable do you feel to developing invasive melanoma sometime in your life? Answer from Not at all vulnerable (0) to Extremely vulnerable (6).

Cognitive measure:

Given the diagnosis of (given label), on a scale of 0–100%, what do you think your chances are of developing an invasive melanoma sometime in your life?

Given the diagnosis of (given label), what do you think your chances are of developing an invasive melanoma, compared to others of your age, gender, and skin colour? Answer from Much lower chance (0) to Much higher chance (6).

Dying from melanoma:

Given the diagnosis of (given label), on a scale of 0-100%, what do you think your chances are of dying from melanoma?

6. The referencing of the manuscript is incomplete. The reference list attached extends to reference 26. However, in the tables, references continue on to reference 31.

Response and Change: This has been corrected.

Reviewer 2

1. This is a topic close to my heart. Especially since the (incredible) decision as made to increase excision margins for MIS to 10 mm. So I have written a bit of a tome.
In summary 'good idea but has flaws and could be a lot better'.

Response: Thank you for your supportive comments and helpful suggestions. We hope that our revisions address your concerns.

2. When I read the article lots of questions sprang to mind. some were answered in the questionaire reading. You need to address these in the article. For example your idea of 'self examination' is not communicated very well in the article. In the hypothetical they are taught self exam. In the primary and secondary outcomes measures you offer them a very sophisticated telemedicine service. Not widely available. This is confusing.

Response: We have expanded the rationale for both co-primary outcomes, including the concept of patient-led surveillance/ patient-initiated follow-up currently restricted to trial setting, but a potentially more sustainable model of care.
Change (page 12, lines 212-226):

"The first co-primary outcome on surgical management choice reflects recent retrospective analyses that have found that narrower margins are likely to be as safe as the margins currently recommended in guidelines in small melanoma in situ. Indeed very narrow histological clearance (≥1mm) appears to be safe for melanoma in situ of the trunk and limbs. The new MPATH-Dx V2.0 melanocytic lesion classification scheme recommends that provided margins are not involved, clinicians may consider not re-excising class II lesions – which includes melanoma in-situ. The second co-primary outcome on follow-up management choice centres around patient-led surveillance (also called patient-initiated follow-up) as an alternative model of follow-up for cancer survivors to routinely scheduled clinic appointments. Among people diagnosed and treated for early stage melanoma, patient-led surveillance is being evaluated in the MEL-SELF randomized controlled trial. Here, this model of care includes: training in self-skin examination, digital technologies to record and take images of concerning lesions (using a mobile dermatoscope), online system for submitting images for remote review by a dermatologist, and advice on whether urgent clinical review may be needed (teledermatology)."

3. In the abstract you state " but existing terminology makes it difficult for patients to distinguish these from higher risk invasive melanomas." This is true but as importantly does this not apply to doctors? The clinicians enrol patients in short term digital dermoscopic imaging trying to capture these lesions, they get histopath reviewed if the report does not read MIS, the pathologists order an array of extra stains and come up with diagnoses such as 'best regarded as MIS" or "evolving MIS" etc, Clinicians then follow the guidelines and do 10 mm margins where they used to do 5 or when the histopath report reads '3 mm clear histological margin' they re-operate to get 5 mm. So I think in fairness you need to discuss this aspect (I think your study is a great idea and reflects local practice here. We tell them 'this lesion is harmless, the trip here for a skin check is more dangerous.).

Response: Thank you for this important comment. We agree that clinicians are also influenced by the MIS label, and are an important group for further research.

Change (pages 4-5, lines 78-82):

- "The study does not explore the potential for recalibration of diagnostic thresholds using existing labels, the impact of diagnostic label on clinician's management decisions, and the impact of providing detailed risk information (and whether this may modify diagnostic label effects). These are important areas for future research"

4. Will you give them an idea of what 'low risk" means?

Response: We will not provide further explanation of what low-risk means. A further area for research could be to examine the effects of providing detailed risk information, and whether this modifies diagnostic label effects.

Change (pages 11, lines 202-203): "We will not provide further explanation of what low-risk means."
Change (pages 4-5, lines 78-82):

- "The study does not explore the potential for recalibration of diagnostic thresholds using existing labels, the impact of diagnostic label on clinician's management decisions, and the impact of providing detailed risk information (and whether this may modify diagnostic label effects). These are important areas for future research."

5. As the study group has no 'skin in the game' i.e. they are not MIS patients will your results help us much?

Response: We deliberatively excluded people with a prior melanoma diagnosis (including MIS) to prevent prior experiences influencing their responses. Although the scenario is hypothetical (they have not actually had a MIS diagnosis), the scenario is a realistic one that they may experience (given the high rate of MIS diagnosis in Australia).

Change (pages 4-5, lines 78-82):

- "The study does not explore the potential for recalibration of diagnostic thresholds using existing labels, the impact of diagnostic label on clinician's management decisions, and the impact of providing detailed risk information (and whether this may modify diagnostic label effects). These are important areas for future research."

6. I wonder what a treating clinician would do if called upon to treat a patient way outside of guidelines . where I work the melanoma guidelines are regarded as Holy Writ by some.

Response: We agree. Please see response to comment #3 above.

7. Where did 0.5 mm come from as an adequate margin? Why not 1 or 2 mm, "clear" or 'well clear"? you should explain that.

Response and Change: This was a typo and should have been 5mm. We have provided more text explaining the rationale for this in the text for the co-primary outcome on surgical management (see changes in response to #2 above).

8. Also how much greater than 0.5 mm was the re excision to achieve? Do you mean 'usual treatment as per the guidelines'? If so might be better to state that.

Response and Change: Please see changes in response to #2 above.

9. Will the choice of patient led follow up be accompanied by education in same? (the evidence re skin self surveillance is pretty damning in the main after all).

Response and Change: Please see changes in response to #2 above.

10. You state "The randomised design will provide highly relevant evidence on potential impacts of alternative diagnostic labels for melanoma in situ to patients' decision-making and psychological outcomes." Is this true if they do not have 'skin in the game'?
Response and Change: Please see response to #5 above.

11. also don't we need to know if there is a significant body of evidence that a 0.5 mm histological margin is safe? This would likely be true for smaller MIS but would it be true for a largish lentigo maligna? If I was a member of the public told they had an MIS I would immediately google it. This will tell us to do a wide excision. So that simple search will influence your subjects in the real world.

Response: Please see changes in response to #2 above.

12. Won't your survey participants be googling the topic? If some do and some do not that might influence their answers?

Response: Although this is a theoretical concern, we have found that participants in these online studies generally complete the survey in a timely fashion without spending time googling their answers. As a data cleaning step, we will exclude participants who completed the survey very quickly, or very slowly, to ensure that those who legitimately engaged with the survey are included in the analysis.

13. "One possible solution is to consider a new label for melanoma in situ without the word “melanoma”. This might help patients recognize the lower risk of this type of lesion, and help to reduce the potential psychological harm. It may also pave the way for the de-escalation of treatment and surveillance."

I agree with your introduction. I work in a region with the highest incidence of melanoma in the world. MIS (real or imagined) abound. Doctors are a large part of this issue. Very common for the patient to never have been told (or at least not know) what their diagnosis of MIS actually means. Nor do they understand why a wide excision is advised i.e. life saving v reduced local recurrence. For this study to have greater relevance why not survey doctors involved in MIS management and see what they would do? after all as it stands very few clinicians will go outside guidelines and actually tell a patient they have the alternative diagnostic label and management plan. I recognise you surveyed clinician coinvestigators but what will the great mass of doctors looking after MIS make of these labels/management options? In australia this is largely done by GPs. Dermatopathologists were surveyed but they are not called upon to make actual management decisions in MIS. Very few MIS end up in the hands of surgical or radiation oncologists.I think to have more veritas you would want to know what GPs/Dermos/Plastics/General surgeons think. Interesting to know and will improve the impact of your paper.

You even state "Alternative label(s) need to be acceptable to both patients and clinicians, and convey the low, but not zero, risk of future invasive melanoma." To me this study is of interest but nobody would use it to inform practice. That is fine of course as this is an important discussion. Management decisions are a 'consensus' between patient and clinician. so expanding your survey to clinicians will add to the impact. Easily done.

Response: We agree that this is an important area for future research (please see response to #3).

14. "patients will be excluded if they have a history of melanoma.' I understand the rationale but it detracts from your study. You include people "40 years or older, understand written English, and reside in Australia." So what if you have a representation of people at very low risk of melanoma i.e. dark skinned ? Will they give the same weight to the hypothetical as red heads with sun damage? What about a patient who has never had melanoma but has a strong family history of same? Or one who has had keratinocyte cancer in the past etc? What about patients who are already 'skin aware' and getting checks despite having no risk factors (heaps of them out there). the point being you are excluding melanoma patients so why not these groups? I would have thought people who have had an MIS would be of equal interest as people at high risk of same?

In the survey will the terms ' melanocytic', 'neoplasm', 'low risk', 'in situ' be defined? Your participants will likely 'google' these. Might effect their answers if they do?

If I was designing this I would confine it to people who have a real/perceived risk of developing melanoma. I recognise you will be collecting this data but would seem logical to survey people with 'skin in the game'.

Response: We are collecting data on melanoma risk factors including those mentioned and will use these to calculate participants' future melanoma risk. We are excluding people with a past melanoma diagnosis for the reasons outlined in response to #5.

15. "Secondary outcomes are: diagnosis anxiety, perceived lifetime risk of invasive melanoma, perceived lifetime risk of dying from melanoma, management choice anxiety, and open-text explanation of management choices (free text input)." They will google this won't they? In other words will they make the assessment based on the information the question provides or do what people do in real life? Either you need to prevent them from doing this in some way (makes it artificial) or ask the question 'did you do your own research'?

Response: Please see response to #12 above.

16. "The analysis will focus on assessing the impact of different diagnostic labels for melanoma in situ on participants' psychological responses and healthcare decisions." This is a bit artificial (I recognise it has to be). A patient will generally make the decision the doctor tells them to make. With your three options the major determinant ill be what their clinician thinks. The commonest thing we hear when presenting management options is 'what would you do?'. The next is 'I will do what you tell me to do.'

Response: We agree that the clinician recommendation is a major determinant of the patient choice of management. To neutralise this effect, we reworded these questions to make it clear that their clinician is not making a recommendation either way.

Change (Supplement, pages 16-17):

Your doctor continues: "You now need to decide whether you would like us to do further surgery to remove more normal skin from around the scar, or whether you would prefer no further surgery at this time. I recommend either of these options as a reasonable choice and will organise whichever you prefer."

Given the diagnosis of (given label), which of these surgery management options would you choose?

"Your doctor further explains that there are also different options for follow-up: "You need to also decide whether you would like to book in for regular skin checks with me every 6 months, or you would like us to teach you how to check your skin yourself (with tele-dermatologist support) and book in with me only if needed. Again, I recommend either option as a reasonable choice, and will organise whichever one you prefer."

Given the diagnosis of (given label), which of these follow-up management options would you choose?

17. Maybe a better way to field the choices is for them to read an advice from the doctor which says 'You have LABEL HERE. In this situation my advice is WIDE EXCISION/NO FURTHER TREATMENT. YOUR FOLLOW UP SHOULD BE. xyz.. YOUR OTHER OPTIONS ARE A or b. I would consider them reasonable but they have these disadvantages....."

Response: We agree that this would be another way to test the effects of the diagnostic label in the context of strong clinician recommendation that is likely the norm, in reality. However, our current study design also tests the label effects, and additionally by removing the clinician recommendation it also allows patient preference to be measured.

18. In the hypothetical the doctor leaves it up to the patient to pick an option with the statement 'I reccommend any of these options as reasonable'.. Is that realistic? I think in the main doctors have pretty strong views on what is the 'right way forward' and equally patients come to us for specific advice.

Response: Although doctors may usually recommend one option as preferred, there are often scenarios where they may not have a clear recommendation for the patient (albeit not necessarily related to melanoma management). Our scenario allows us to more directly measure patient preference, uninfluenced by clinician recommendation.

19. You do not mention the 0.5 mm histology clearance in the hypothetical as far as I can see?

Response: We now mention the 3mm clearance and further surgery for 5 mm clearance in the scenario and management question (the 0.5mm was a typo).

Change (Supplement, Page 14 and 16):

"You are at the doctor (GP) after you recently had a small surgery done to remove one of your moles.

The doctor has the pathology test results and says: “We found a (given label). We removed it all, and also 3mm of normal skin around the (given label)."

Given the diagnosis of (given label), which of these surgery management options would you choose?

> Further surgery to remove more normal skin around the scar (so that the distance from the margins to the (given label) is greater than 5 mm)
>
> Or
>
> No further surgery”

20. After all no patient makes such an important decision based on the diagnosis alone. they always seek more information and guidance, do their own research etc.
Response: We agree that the scenario is hypothetical and state this as a limitation. See also responses to #17 and #18 above.

21. "Two authors have lived experience of a melanoma diagnosis" Were these of MIS only? That is likely relevant.
Response: One patient author had MIS and one had a thin stage I invasive melanoma

Change (pages 15, lines 282-283): “Two authors have lived experience of a melanoma diagnosis (one had MIS and one had a thin stage I invasive melanoma),”

22. Can you make this claim from this study? "The potential significance of this study lies in its ability to impact clinical practice and policy by identifying alternative diagnostic labels for melanoma in-situ that are acceptable to patients, pathologists, and treating clinicians." You are only surveying members of the public. We can make no inferences as to what pathologists and clinicians think of these without surveying them can we?

Response and Change: This text has been removed.

23. "Alternative labeling of melanoma in-situ may better inform patients about the low level of risk....." The big thing is likely not the label but the management. We tell patients 'you have had an MIS. The lesion is all out. the risk from this is minimal, in fact recent studies have shown that your chances of being alive in 5 years are higher than those who have not had a MIS. However we need to take an extra 10 mm margin." After all with a BCC, IEC, low risk SCC, KA we just treat it and say 'that is it no further treatment needed.' similar issue i.e. a negligible risk lesion but a very different management paradigm.
Response: We believe that both the label and the management are important. We agree that there is a need for better communication about risk, and the potential impact of this is an area for future research (see response to #4 above).

24. In the article you state the study is restricted to 40 years and older. In your table it states 18 and older to be legible.
Response: Thank you, this has been corrected.

25. In the article you talk about 'self examination' but in the questionaire it turns out you are going to get them involved in a telemedicine service. You need to make it clear in the article what you mean by self examination , this is not what most people would consider self examination is it?

Response and Change: Please see response to #2 above.

26. For me this is a good idea but could be a lot better. It will not inform future practice but it will support/inform further research into changing the management of MIS. I think merely knowing what a theoretical patient thinks is not nearly as useful as what their treating doctor would also think.

Response and Change: Please see response to #3 and #4 above.

27. Any observer of the clinical 'scene' will recognise that there is a large body of clinicians and pathologists who agonise over the diagnosis of MIS. We regularly see reports where the pathologist has sought multiple opinions, ordered an array of stains etc to distinguish 'severe atypia/high grade dysplasia' from MIS. Often pathologists will not give a firm diagnosis but say 'on balance best regraded as MIS'. Casual observers of the dermoscopy web sites will see regular tales of clinicians demanding more opinions/deepers until a 'correct' diagnosis of MIS is rendered. So I would suggest you survey all involved in MIS (patients, clinicians and pathologists) re this. Preferably as part of the study or in future.

Response and Change: We agree. Please see response #3 above.

28. The article is a bit confusing/misleading at times, see above.

Response and Change: We believe that the revisions in response to the helpful comments from Editors and Reviewers have improved the article. We hope that you now do not find it confusing or misleading.

VERSION 2 - REVIEW

**Reviewer**	**2**
**Name**	**Muir, James**
**Affiliation**	**The University of Queensland**
**Date**	**07-Nov-2024**
**COI**	

---

you still have 0.5 mm margin? I thought in your response you were changing this?

Melanoma is common here but not that common, most Australians will never get one. I don't think we can claim this paper approaches being 'realistic' does it? to do that we would need to do this on actual patients who have had a lesion excised.

I think you should say 'our research may guide future studies on patients who have undergone excision of suspect skin lesions where melanoma was proffered as a possible diagnosis.'

Does the label make patients worry re death or is it the fact their doctor has not explained their diagnosis to them? The latter is the crucial thing in my experience.

Will arm 1 and 3 understand what 'in situ' means? will this be explained? You may find some of the surveyed do not understand 'melanocytic' or 'neoplasm'. Some might interpret that to mean cancer (it is not rare to do so). Others might interpret it to mean harmless. The consistent message to all 3 groups should really be 'you have an almost certainly 'harmless' cancer'. To reflect life we would need to state in arm 1 that thier MIS is low risk don't we? or at least detail what in situ means?

You state enrolment will be from 1/7/24 to 1/8/24? Aren't we past that?

I still think the lack of skin in the game makes this paper pretty "soft". Best it can do is use it to support doing this in actual patients.

VERSION 2 - AUTHOR RESPONSE

Reviewer 1's Comments

1. you still have 0.5 mm margin? I thought in your response you were changing this?

Response and Changes: Thank you for picking this up. We have now corrected this to 5mm margin throughout the manuscript.

2. Melanoma is common here but not that common, most Australians will never get one. I don't think we can claim this paper approaches being 'realistic' does it? to do that we would need to do this on actual patients who have had a lesion excised. I think you should say 'our research may guide future studies on patients who have undergone excision of suspect skin lesions where melanoma was proffered as a possible diagnosis.'

Response and change: Thank you for your comments and suggestions. We agree a study involving actual patients who have had a lesion excised could be a worthwhile focus for future research. In this study, we deliberately excluded participants with a prior melanoma diagnosis to prevent any influence of previous experiences on their responses. We acknowledge that melanoma is not something that most Australians will have. Still, we think the hypothetical scenario reflects a situation that participants could face, given the high melanoma and skin cancer rate in Australia. We have changed the wording of this limitation in response to your comment (see above on point 2).

3. Does the label make patients worry re death or is it the fact their doctor has not explained their diagnosis to them? The latter is the crucial thing in my experience.

Response: Thank you for your comment. We agree that the clinicians' explanation is a major determinant of how a patient feels about their diagnosis, and have recommended this as an area for future research. In this study, however, we aimed to isolate the effect of diagnostic labels on patient preferences by having a neutral stance from the clinician. In our hypothetical scenario, we detail how the diagnosis and its recommendation are communicated to the patient by a doctor (see Supplement, pages 16-17). The aim was to then explore the impact of different labels on patients' management choices and anxiety levels.

4. Will arm 1 and 3 understand what 'in situ' means? will this be explained? You may find some of the surveyed do not understand 'melanocytic' or 'neoplasm'. Some might interpret that to mean cancer (it is not rare to do so). Others might interpret it to mean

harmless. The consistent message to all 3 groups should really be 'you have an almost certainly 'harmless' cancer'. To reflect life we would need to state in arm 1 that thier MIS is low risk don't we? or at least detail what in situ means?

Response: Thank you for your comment. We chose not to explain terms like "in situ" directly in the study, as we aim to explore how participants' health literacy and education levels might influence their responses to the intervention. We have implied that the scenario involves a small surgery and provided participants with the option to choose the management and surgery options, which we think will offer reassurance about the low-risk nature of the diagnosis. The choice of alternative diagnostic labels was made in consultation with both 6 Patient/Public co-investigators and 9 international Clinician co-investigators to ensure clarity and relevance (see page 10).

5. You state enrolment will be from 1/7/24 to 1/8/24? Aren't we past that?

Response: This protocol paper was submitted on 03/06/24, before we commenced recruitment. Data collection is now complete.

6. I still think the lack of skin in the game makes this paper pretty "soft". Best it can do is use it to support doing this in actual patients.

Response: We acknowledge this limitation. We believe this study is an important first step in investigate the potential for alternative labels. Future research could investigate alternative labels actual patients and clinicians based on this study.
